# Editorial Special Issue „Leadership in a Digital World”

**DOI:** 10.1365/s42681-019-00004-y

**Published:** 2020-09-24

**Authors:** Werner G. Faix

**Affiliations:** Berlin, Germany

Digital transformation is a keyword that not only dominates the media, but is also discussed in the research departments of companies, by politicians and in society as a whole. But this is just one of the many challenges we face. The currently ongoing coronavirus pandemic as well as continuing globalization, sustainability issues and the possibilities of biological transformation and its opportunities for totaly new business models are urgent issues that we need to address.

The changes associated with this transformation are partly connected with concerns or fears, because the effects are perceivable at all levels of society and are by no means always trivial and understandable. This applies, for example, to transformation processes in the world of work that go hand in hand with digitization, not least due to the possibilities offered by artificial intelligence. To master these challenges, “management” is not enough, while “leadership” is gaining in importance.

In times of change, leaders must above all fulfil two tasks: On the one hand, they must be able to draw an attractive picture of the future. They must be able to convince even the skeptics to follow the—often strenuous—path and find it rewarding. On the other hand, leaders must inspire themselves and others to achieve challenging goals. Because it is not enough just to be ready for the future. It is not enough to let “the others” implement digitalization or other changes. In order to live together in an attractive future at the end of the day, one thing is still needed first and foremost: innovations!

But incremental innovations cannot make the necessary progress. For example, a marginal improvement in the injection nozzle of a combustion engine will not do justice to the finite nature of fossil fuels. Only radical and disruptive innovations will make it possible to develop solutions for current and future challenges. Innovation should not be understood as a process that takes place once every 10–20 years in order to have done enough for advancement. Innovation is a constant process, as are changes. And anyone who tries to meet changes that have already happened with innovations that are then urgently needed will fail. The present situation is appropriately summarized in the maxim: “If you innovate in time, you have in the future. Those who innovate only in the future do not have much time left.” At this point it must be mentioned that innovation can never be measured by the number of patents. Many patents are unused. Innovation only happens when a good idea becomes value-adding reality.

The required innovations must also be generated at all levels and in all departments of an agile organization. Focusing on research and development is not enough. In particular, leaders must face up to change and actively shape the future. They act as an example to give those employees with value-adding ideas a leadership role in the innovation process.

The urgency of changes is thus unmistakable, as is the enormous influence that digitization has on our lives. Nevertheless, there is still relatively little knowledge of the connections between digital transformation, leadership and work design in the current state of research. Therefore, the special edition of “Leadership, Education, Personality: An Interdisciplinary Journal” focuses on the topic ”Leadership in a Digital World”. The two terms “digitization” and “leadership” are highly interdisciplinary and therefore require comprehensive consideration from various scientific perspectives: Basic research as well as applied research—without restrictions regarding methodological procedures—can provide valuable insights.

In addition, it should also be mentioned that the involvement of the Western world is not sufficient to develop solutions for the challenges of the future. Global awareness of the changes and the need for innovation must be achieved. Therefore, we are particularly pleased to have an article in this special issue that looks at the progress of digitization in Kenya.

Editor-in-Chief

Prof. Dr. Dr. h.c. Werner G. Faix

Die digitale Transformation ist ein Schlagwort, das nicht nur die Medien dominiert, sondern auch in Entwicklungsabteilungen von Unternehmen, von Politikern und in der gesamten Gesellschaft diskutiert wird. Doch es handelt sich dabei nur um eine von vielfältigen Herausforderungen, der wir gegenüberstehen. Die aktuell stattfindende Corona-Pandemie als auch die anhaltende Globalisierung, Fragen zur Nachhaltigkeit und die Möglichkeiten der Biologischen Transformation und daraus folgend die Gestaltung völlig neuer Geschäftsmodelle sind dringende Anliegen, denen wir uns widmen müssen.

Die mit dem Wandel einhergehenden Veränderungen, sind zum Teil mit Sorgen oder Ängsten verbunden, denn die Auswirkungen sind auf allen gesellschaftlichen Ebenen spürbar und längst nicht immer trivial und begreifbar. Dies trifft zum Beispiel auf Transformationprozesse in der Arbeitswelt zu, die mit der Digitalisierung einher gehen, nicht zuletzt durch die Möglichkeiten der künstlichen Intelligenz. Um diese Herausforderungen zu meistern, reicht “Management” nicht aus, während “Führung” an Bedeutung gewinnt.

Führungskräfte müssen in Zeiten des Umbruchs vor allem zwei Aufgaben erfüllen: Zum einen müssen sie es schaffen, ein attraktives Bild der Zukunft zu zeichnen. Sie müssen es schaffen, auch die Skeptiker zu überzeugen, den – oft mühsamen – Weg zu beschreiten und diesen für lohnend zu empfinden. Zum anderen müssen Führungskräfte sich selbst und andere Menschen dazu inspirieren, anspruchsvolle Ziele zu erreichen. Denn es reicht nicht aus, nur bereit für die Zukunft zu sein. Es reicht nicht aus „die anderen“die Digitalisierung oder andere Veränderungen umsetzen zu lassen. Um am Ende gemeinsam in einer attraktiven Zukunft zu leben braucht es, nach wie vor, vor allem eins: Innovationen!

Dabei können inkrementelle Innovationen nicht den notwenigen Fortschritt erzielen. Beispielsweise wird eine marginale Verbesserung der Einspritzdüse eines Verbrennungsmotors der Endlichkeit fossiler Brennstoffe nicht gerecht. Nur mit radikalen und disruptiven Innovationen wird es gelingen, für aktuelle und für zukünftige Herausforderungen Lösungen zu entwickeln. Dabei ist Innovation nicht als ein Vorgang zu verstehen, der einmal alle 10 bis 20 Jahre erfolgt, um dann wieder ausreichend für den Fortschritt getan zu haben. Innovation ist ein stetiger Prozess, so wie die Veränderungen auch. Und wer versucht bereits erfolgten Veränderungen mit dann eilends notwendigen Innovationen zu begegnen, wird scheitern. Die vorliegende Situation wird angemessen zusammengefasst in dem Leitspruch: „Wer innoviert in der Zeit, der hat in der Zukunft. Wer erst in der Zukunft innoviert, dem bleibt nicht mehr viel Zeit“. An dieser Stelle sollte erwähnt sein, dass Innovation keinesfalls an Patenten gemessen werden kann. Viele Patente fristen ein ungenutztes Dasein. Innovation geschieht erst dann, wenn eine gute Idee wertschöpfende Wirklichkeit wird.

Die erforderlichen Innovationen müssen zudem auf allen Ebenen und in allen Abteilungen einer agilen Organisation generiert werden. Den Fokus auf Forschung und Entwicklung zu setzen ist nicht ausreichend. Insbesondere die Führungskräfte müssen sich dem Wandel stellen und die Zukunft aktiv gestalten. Dabei fungieren sie als Vorbild, um im Innovationsprozess denjenigen Mitarbeitern mit wertschöpfenden Ideen selbst eine Leadership-Rolle zukommen zu lassen.

Die Dringlichkeit der Veränderungen ist somit unverkennbar, sowie auch der enorme Einfluss den unter anderem die Digitalisierung auf unser Leben hat. Dennoch findet sich im aktuellen Forschungsstand noch relativ wenig Wissen über die Zusammenhänge zwischen digitaler Transformation, Führung und Arbeitsgestaltung. Daher konzentriert sich die Sonderausgabe von “Leadership, Education, Personality: An Interdisciplinary Journal” auf das Thema “Leadership in a Digital World”. Dabei sind die beiden Begriffe Digitalisierung und Leadership im höchsten Maße interdisziplinär und erfordern daher eine umfassende Betrachtung aus diversen wissenschaftlichen Blickwinkeln: Grundlagenforschung wie angewandte Grundlagenforschung – ohne Einschränkung bezüglich methodischer Verfahren – können wertvolle Einblicke liefern.

Darüber hinaus sei noch erwähnt: Um Lösungskonzepte für die Herausforderungen der Zukunft zu entwickeln, reicht Engagement der westlichen Welt nicht aus. Global muss ein Bewusstsein für die Veränderungen und für die Notwendigkeit von Innovationen erreicht werden. Daher freuen wir uns besonders über einen Beitrag in dieser Sonderausgabe, der das Voranschreiten der Digitalisierung in Hinblick auf Kenia betrachtet.

Editor-in-Chief

Prof. Dr. Dr. h.c. Werner G. Faix

